# Earliest details of dermatology by Ayurveda^☆^^☆☆^

**DOI:** 10.1016/j.abd.2021.05.002

**Published:** 2021-07-15

**Authors:** Rashmi TM, Sathish HS

**Affiliations:** a Department of Shalya Tantra, TMAE’s Society Ayurvedic Medical College, Shimoga, Karnataka, India; b Department of Shalya Tantra, TMAE’S Ayurvedic Medical College, Shimoga, Karnataka, India

Dear Editor,

I read the article entitled “History of dermatology: the study of skin diseases over the centuries”,^1^ which is, astonishingly, the earliest descriptions of skin and its disorders detailed in the Ancient Indian Vedic texts, especially in Ayurvedic treatises have been unfound.^2^, ^3^ Ayurveda, the Science of life, is the first treatise that contains numerous evidence of the origin of dermatology. *Sushruta* mentions seven layers of skin with a specific thickness and also dermatological disorders affecting each layer.^4^ (Table 1). The layers of *twak* are, *Avabhasini, Lohita*, *Shweta*, *Tamra*, *Vedini*, *Rohini,* and *Mamsadhara*.^5^, ^6^

**Table 1:** Seven layers of skin with specific thickness.

Sl. nº	Layers of *Twak* (Skin)	Diseases affecting the layer
1	*Avabhasini*	*Sidhma* (Pityriasis Vesicular), *Padmakantaka* (Papilloma)
2	*Lohita*	*Tilakalaka* (Non elevated Mole), *Nyaccha* (Naevi), *Vyanga* (Freckle)
3	*Shweta*	*Ajagallika* (Molluscum Contagiosum), *Charmadala* (Atopic dermatitis), *Mashaka* (Raised Mole)
4	*Tamra*	*Kilasa kushta* (A form of Leucoderma)
5	*Vedini*	*Kushta* (Leprosy and other serious skin disorders), *Visarpa* (Erysipelas)
6	*Rohini*	*Granthi* (Cyst), *Apachi* (Cervical Lymphadenitis), *Arbuda* (Neoplasm), *Shleepada* (Elephantiasis), *Galaganda* (Goitre)
7	*Mamsadhara*	*Bhagadara* (Fistula), *Vidradhi* (Abscess)

Ayurveda designates Dermatological disorders as *Kushta. Kushta*, is the term assigned to the skin disorders in Ayurvedic texts, it includes various forms of pathologies of the integument system. The etiological factors for skin disorders are classified as physical, physiological, hereditary, and psychological,^7^ and there is one more segment of etiology which basically talks about sinful acts of an individual resulting in the development of pathological manifestation in the skin, the veracity of this cause has yet not been researched. Grossly, the disorders are grouped into two as *Mahakushta* (Skin disorders with a major imbalance of dosha or bodily humor with a deeper level of pathological invasion) and *Kshudra kushta* (skin disorders with less degree of vitiation of *dosha*).^8^, ^9^ The dreaded skin ailment, Leprosy, its etiopathogenesis, complications, and treatment modalities are described in detail in *Sushruta’s* treatise.^10^ Descriptions of Leprosy can be traced from all the ancient manuscripts of Vedic and post Vedic era, and numerous indigenous treatment modalities and certain other treatment modalities of religious and spiritual importance are also been described.^11^ Plenty of Ayurvedic practitioners are successfully treating skin disorders by adhering to the principles of Ayurveda as obtained through texts.

## Financial support

None declared.

## Authors’ contributions

Rashmi TM: Approval of the final version of the manuscript; drafting and editing of the manuscript.

Sathish HS: Approval of the final version of the manuscript; drafting and editing of the manuscript.

## Conflicts of interest

None declared.
